# Metagenomic data on *Anadara granosa* associated bacterial communities using culture dependent approaches and 16S rRNA sequencing

**DOI:** 10.1016/j.dib.2018.05.052

**Published:** 2018-05-23

**Authors:** Kamarul Zaman Zarkasi, Mardani Abdul Halim, Teh Faridah Nazari, Feizal Daud

**Affiliations:** a School of Biological Sciences, Universiti Sains Malaysia, Penang, Malaysia; b School of Natural and Environmental Sciences, Newcastle University, Newcastle upon Tyne, United Kingdom; c Faculty of Plantation and Agrotechnology, Universiti Teknologi MARA, Shah Alam, Selangor, Malaysia

## Abstract

This article contains data on the bacterial communities and its diversity associated with *Anadara granosa*. The *A. granosa* samples were obtained from two major estuaries in Penang, Malaysia using a culture dependent and 16S rRNA Illumina sequencing approaches. *A. granosa*, a commercial blood cockles and popular seafoods, is fragile to the surrounding environments. Thus, our research focused to better understand the bacterial communities and it diversity in the *A. granosa*, as well as on the generation of a metagenomic library from *A. granosa* to further understanding on it diversity. The bacteria Vibrionaceae (34.1%) was predominant in the *A. granosa* from both environments followed by Enterobacteriaceae (33.3%) and Bacillaceae (16.75%). *Vibrio* sp., *Klebsiella* sp., and *Bacillus subtilis* were the most abundant species present. The data generated in this research is the first metagenomic examination of *A. granosa* and will provide as a baseline to understand the bacterial communities associated with *A. granosa* and its surrounding natural environments.

**Specifications Table**

**Table:** 

Subject area	Biology
More specific subject area	Microbiome.
Type of data	Text Files and Figures.
How data was acquired	Culture-dependent analysis and the16S rRNA genes using the Illumina sequencing-by-synthesis method (MiSeq platform).
Data format	Raw and analyzed.
Experimental factors	Bacterial genomic DNA was extracted and used as a template to amplify the V3-V4 region of the 16S rRNA gene. The amplicons were molecularly barcoded, pooled, and sequenced by Illumina MiSeq.
Experimental features	Culture-dependent analysis data was constructed using general methods, and sequencing of the 16S rRNA gene amplicon using the Illumina MiSeq platform were performed from sample obtained from the *Anadara granosa*.
Data source location	The samples were collected in Penang, Malaysia.
Data accessibility	Data is within this article and have been uploaded to Zenodo (https://doi.org/10.5281/zenodo.1171157).

**Value of the data**

• This project presents the diversity of bacteria communities of *A. granosa* by using culture-dependent and 16S rRNA gene sequencing.
• This project expended our knowledge of bacterial communities and it diversity in the *A. granosa* and surrounding environments.
• These data are the first generated using 16S rRNA genes Illumina sequencing of *A. granosa* in Malaysia and gaining a better understanding of the *A. granosa* bacterial community diversity.

## 1 Data

**Fig. 1:**
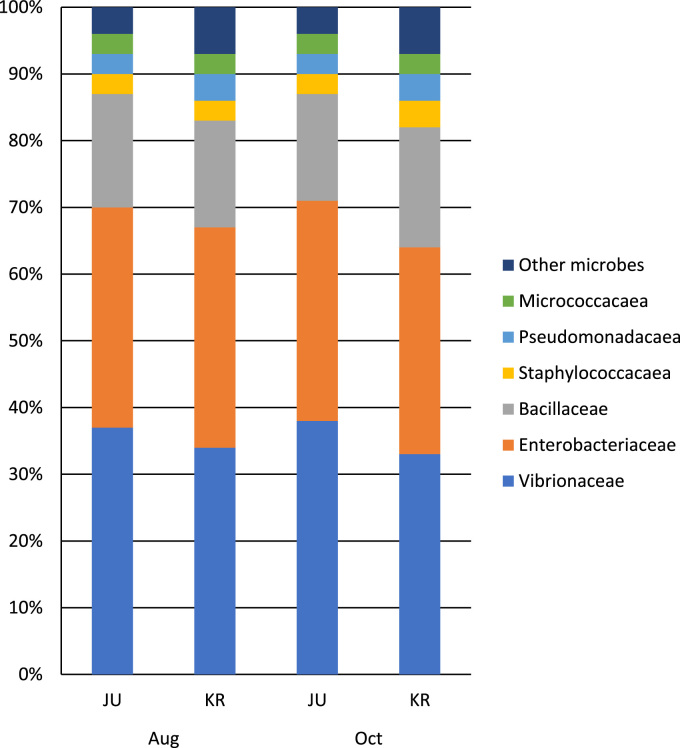
Average composition of bacteria from all samples by cultivation techniques.

**Fig. 2:**
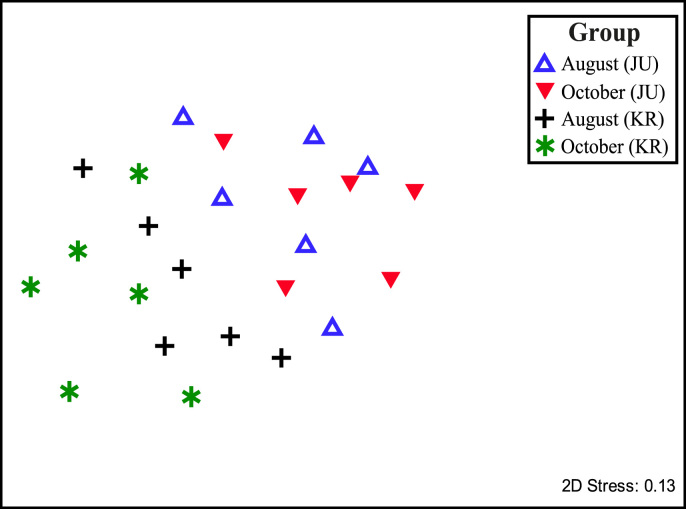
MDS plots showing the bacterial community diversity associated with *Anadara granosa* and the factor of sampling location.

The bacteria׳s 16S rRNA sequencing such as metagenomics increasingly important for agriculture, aquaculture, ecology and environmental studies for the past few years [1]. This methodology become popular mainly to understand the bacterial community diversity in the environments, animals and plants. Culture dependent approach is a traditional way to understand the bacterial communities and it allows to access culturable bacteria in the most habitats [2], while culture independent approach such as 16S rRNA sequencing allows to identify unculturable bacteria that may count >90% of the bacterial population, who are may unnoticed and not yet identified [3]. This methodology will be allowed to further study, understanding and manipulating bacterial communities present in the environment, animals and plants. In this project, culture dependent technique was conducted and 16S rRNA Illumina sequencing were performed. Fig. 1 shows the bacterial community structure from the culture dependent technique. Fig. 2 and Table 1 provide overview of the bacterial community diversity using 16S rRNA Illumina sequencing and the factor of sampling location. The data is interesting for understanding the bacterial community diversity associated with *Anadara granosa*, and how the factor such as sampling location may influence the bacterial community and it dynamic. This study shows that sampling location and environmental factors shapes the bacterial community and its diversity.

**Table 1:** Identities of the five most abundant OTUs in the bacterial communities.

**Abundance order**	**Taxa (% of abundance)**	
	**JU**	**KR**
1	*Vibrio* sp. (12.2%)	*Vibrio* sp. (12.5%)
2	*Klebsiella pneumoniae* (10.1%)	*Bacillus subtilis* (10.3%)
3	*Vibrio parahaemolyticus* (9.9%)	*Klebsiella* sp. (9.4%)
4	*E. coli* (9.9%)	*Enterobacter* sp. (8.7%)
5	*Bacillus subtilis* (8.5%)	*E. coli* (8.0%)

## 2 Experimental design, materials and methods

### 2.1 Samples collection

The *A. granosa* samples were collected from coastal area in Penang, Malaysia in August and October 2017 at two different sampling locations. The two sampling locations where samples collected are Juru (L1) and Transkrian (L2). The sampling location of L1 is near to the industrial zone, where the source of pollution problems has been identified [4], while for sampling location of L2, which is near to the agriculture zone and residential area. Both sampling locations were chosen because we want to compare the location factor that may shape and influence the bacterial community diversity associated with *A. granosa*. Total 24 samples (12 samples per sampling location) were randomly collected.

### 2.2 Microbial cultivation and enumeration

The *A. granosa* samples were grouped into two different groups according to their sampling location (L1 and L2). The samples were then cleaned under running tap water with a brush to remove any sand, debris and mud on the blood cockle׳s shell. Then the raw *A. granosa* were aseptically shucked using a sterile knife with intact bodies and liquor placed and pooled into a sterilized filter blender bag. The bag was massaged through by hand for one minute to separate the excess shell from the liquor and intact bodies. Then, all samples were transferred into a new full filter blender bag to remove remaining shells. A liquor of 3% sea salt peptone water (around 450 ml) was added and homogenised for two minutes [5] Samples (5 ml) were taken and processed for microbial cultivation and enumeration, as well as DNA extraction. Serial dilutions were performed and spread onto three types of agar media; Brain-Heart Infusion (BHI) Agar with 3% Sea salt, Marine Agar with 3% sea salt and thiosulfate-citrate-bile salts-sucrose (TCBS) agar [1]. Plates were incubated according to aerobic and anaerobic atmosphere (using AnaeroGen kit by Oxoid) at 25 °C for 24–72 h respectively. After 24–72 h of incubation, all plates were read and examined by the standard plate count method, and pure colonies were isolated. Then, pure colony characterization and identification were observed and identified.

### 2.3 DNA extraction

DNA extraction was performed using the QIAamp DNA Mini Kit (Qiagen Sciences, Germantown, MD, US) following the manufacturer׳s instruction and standard protocols [6].

### 2.4 16S rRNA gene Illumina analysis

Sequencing of the 16S rRNA gene amplicon was applied to the 24 samples collected using the Illumina MiSeq platform. Pair-ended PCR amplification of the 16S rRNA gene V3-V4 region was carried out using 341F and 907R primers that possessed 12 bp barcode tags [6]. FASTQ files generated were merged using PEAR [7], these were then trimmed to remove the primer, barcode and adapter regions. The seed sequence for each cluster was then sorted by length and clustered with a 3% divergence cut-off to create centroid clusters. Clusters containing only <2 sequences or <100 bp in length were then removed [7]. Seed sequences were again clustered at a 3% divergence level using USearch to confirm whether any additional clusters appeared. Consensus sequences from these clusters were then accurately obtained using UPARSE [8]. Each consensus sequence and its clustered centroid of reads was then analyzed to remove chimeras utilizing UCHIME in the de novo mode [9]. After chimera removal, each consensus sequence and its centroid cluster were denoised in UCHIME in which base position quality scores of >30 acted as the denoising criterion. Sequence de-replication and OTU demarcation were further performed in USEARCH and UPARSE to yield OTUs that were aligned using MUSCLE [10] and FastTree [11] that infers approximate maximum likelihood phylogenetic trees. OTUs were then classified using the RDP Classifier [12] against the curated GreenGenes 16S rRNA gene database [13] utilizing the December 2017 database update.

### 2.5 Statistical analysis

PRIMER6 and PERMANOVA+ (version 6.1.12 and version 1.0.2; Primer-E, Ivybridge, UK), respectively, were used to conduct analysis of variance (ANOVA) [6] and multidimensional scaling (MDS) [6] to assess the influence of sampling location on community compositions. For this analysis, results of data collected from the MiSeq Illumina-based 16S rRNA gene sequencing were tabulated with the size bins combined across the samples, square root-transformed and a resemblance matrix created by calculation of Braye-Curtis coefficients. The ANOVA-derived significance values were considered significant when *P*<0.05.
